# INTERPROFESSIONAL COLLABORATIVE RESEARCH ASSESSING OLDER ADULT TECHNOLOGY USE (OR NONUSE)

**DOI:** 10.1093/geroni/igac059.2706

**Published:** 2022-12-20

**Authors:** Donna Case, Sheryl Groden, Michelle Sahli, Wendy Tremaine

**Affiliations:** University of Michigan-Flint, Flint, Michigan, United States; University of Michigan-Flint, Flint, Michigan, United States; University of Michigan-Flint, Flint, Michigan, United States; University of Michigan-Flint, Flint, Michigan, United States

## Abstract

This interprofessional study conducted by faculty in Occupational Therapy, Social Work and Public Health explored older adult technology use (or nonuse) by 216 Genesee County Michigan residents. Three professional lenses informed survey development, implementation and data analysis. Occupational therapy emphasized accessibility, how physical or cognitive impairments hinder technology use, and role of technology to complete daily living activities. Social work focused on technology use to facilitate social connectivity, decrease risks for mental health problems, and resource access. Public health explored if technology use or nonuse impacts health. Results indicated that 14% of participants want to learn to obtain transportation (social, health) via the internet, 13% stated they wanted to learn how to use technology to access medical records, 12.5% to attend on-line appointments, 11% reported they would engage in additional technology-based social activities (e.g. communicating with family/friends), 10% to order/refill medications, and 9% of older adults surveyed had difficulty accessing technology due to a physical, cognitive or sensory impairment. Researchers found the questions provided an integrated view of factors influencing older adult use or nonuse of technology and provided a guide for designing collaborative interventions to facilitate older adult access and use of technology to result in positive holistic health outcomes.

